# Corosolic Acid in Hepatocellular Carcinoma: Molecular Properties, Evidence Boundaries, and Development Priorities

**DOI:** 10.3390/molecules31193474

**Published:** 2026-09-29

**Authors:** Shiyi Zhu, Baoping Chen, Jiarui Qu, Wei Wang, Zaiqi Zhang

**Affiliations:** 1 Hengyang Medical College, University of South China, Hengyang 421001, China; zsylvie11@163.com (S.Z.); 18799479998@163.com (J.Q.); 2 Graduate School, Youjiang Medical University for Nationalities, Baise 533000, China; 17680719376@163.com; 3 TCM and Ethnomedicine Innovation & Development International Laboratory, School of Pharmacy, Hunan University of Chinese Medicine, Changsha 410208, China; 4 Hunan Provincial Key Laboratory of Dong Medicine, Hunan University of Medicine, Huaihua 418000, China

**Keywords:** corosolic acid, hepatocellular carcinoma, pentacyclic triterpenoid, natural product, YAP, ferroptosis, pharmacokinetics, drug development

## Abstract

Hepatocellular carcinoma (HCC) is the most common primary liver cancer and remains a major cause of cancer-related death worldwide. Corosolic acid (CA), an ursane-type pentacyclic triterpenoid with antidiabetic, anti-inflammatory, and antitumour activities, has attracted interest as a potential natural compound for HCC treatment. This review critically evaluates CA as a molecular lead for HCC by integrating its physicochemical characteristics with direct preclinical evidence, cross-model hypotheses, and development constraints. Current studies indicate that CA suppresses HCC cell proliferation and migration, disrupts cell-cycle progression, and promotes cell death, with antitumour effects also observed in animal models. These activities have been associated with mitochondrial apoptosis and endoplasmic reticulum stress, Yes-associated protein (YAP)-related growth regulation, increased susceptibility to ferroptosis, and inhibition of vascular endothelial growth factor receptor 2 (VEGFR2)-related migratory signalling. Interestingly, evidence from other tumour, metabolic, inflammatory, and fibrotic models further suggests that CA may influence tumour metabolism, autophagy, the immunosuppressive microenvironment, fibrosis, and angiogenesis. However, these cross-model findings require direct validation in HCC. Although CA shows promising preclinical activity, it should be regarded as a natural lead compound rather than an established HCC therapy. Major unresolved gaps include poorly defined dose–exposure–response relationships, limited validation of the principal mechanisms in clinically relevant HCC models, and insufficient evidence that optimised formulations or combination regimens improve efficacy without increasing toxicity.

## 1. Introduction

### 1.1. Therapeutic Challenges in Hepatocellular Carcinoma and the Rationale for Studying Corosolic Acid

Hepatocellular carcinoma (HCC), the most common primary liver cancer, is characterised by frequent recurrence and substantial tumour heterogeneity and remains a major cause of cancer-related mortality worldwide [1]. Management is stage-dependent and integrates resection, transplantation, ablation, locoregional therapy, and systemic treatment. For unresectable or advanced HCC, systemic therapy has moved beyond single-agent multikinase inhibition to immune-checkpoint-based combinations, including atezolizumab plus bevacizumab and durvalumab plus tremelimumab for suitable patients with preserved liver function [2]. When these combinations are unsuitable, established alternatives include sorafenib, lenvatinib, or durvalumab [2]. Treatment selection must consider liver function, performance status, portal hypertension and bleeding risk, autoimmune disease, comorbidities, and previous treatment [2]. Locoregional planning also remains important because treatment of tumours with an insufficient future liver remnant must balance tumour control against regeneration of normal liver tissue [3,4,5,6]. Metabolic context matters as well because diabetes is a risk factor for liver cancer, and nonalcoholic fatty liver disease is closely associated with HCC [7,8].

Corosolic acid (CA) is an ursane-type pentacyclic triterpenoid found primarily in *Lagerstroemia speciosa* and other medicinal plants. Its reported glucose-lowering, anti-inflammatory, and antitumour activities have prompted increasing interest [9,10,11]. Preclinical studies indicate that CA inhibits liver cancer cell proliferation and colony formation, alters cell-cycle progression, promotes cell death, and reduces migration. Some of these effects have been reproduced in xenograft and orthotopic liver tumour models [3,4,5,6,7,12,13]. These cellular and animal findings support the systematic evaluation of CA but do not establish its clinical efficacy as an HCC treatment [10,11,14]. Within the current treatment landscape, CA is best positioned as a preclinical lead compound rather than an alternative to established therapies. Any future role, potentially as an adjunct to standard treatment, would require reproducible efficacy, achievable systemic and tumour exposure, safety in chronic liver disease, and evidence of compatibility with approved regimens.

### 1.2. Progress in Corosolic Acid Research and Major Evidence Gaps

Research on CA in HCC has evolved from initial cytotoxicity screening to broader investigations of proliferation, cell-cycle regulation, regulated cell death, migration, and in vivo tumour suppression. Reported antiproliferative IC50 values varied with the experimental model and exposure conditions, ranging from 23.5 ± 2.6 μM in HepG2 cells after 48 h to approximately 40–41 μM across several liver cancer cell lines after 24 h [6,15]. Study-specific IC50, EC50, and CC50 values, together with the corresponding treatment conditions, are summarised in Table 1. Reported mechanisms include mitochondrial apoptosis, protein kinase R-like endoplasmic reticulum kinase (PERK)–eukaryotic translation initiation factor 2 alpha (eIF2α)–activating transcription factor 4 (ATF4)-associated stress, homocysteine-inducible endoplasmic reticulum stress protein with ubiquitin-like domain 1 (HERPUD1)-dependent glutathione depletion, and vascular endothelial growth factor receptor 2 (VEGFR2)–proto-oncogene tyrosine-protein kinase Src (Src)–focal adhesion kinase (FAK)–cell division cycle 42 (Cdc42) signalling [3,5,12,16]. The strength of causal evidence varies. Molecular associations, functional perturbation experiments, and direct-binding assays provide different levels of causal support, and no single pathway accounts for all experimental models [3,5,6,7,12,13].

Studies in prostate, lung, gastric, glioblastoma, ovarian, renal, colorectal, and bladder cancer models, as well as metabolic and inflammatory models, implicate phosphoinositide 3-kinase (PI3K)/protein kinase B (AKT)/mechanistic target of rapamycin (mTOR), adenosine monophosphate-activated protein kinase (AMPK), autophagy, nuclear factor kappa B (NF-κB), and signal transducer and activator of transcription 3 (STAT3) [8,10,11,21,22,23,24,25,26,27,28,29,30,31,32,33,34,35,36]. These cross-model findings require direct testing in HCC. Translation is also limited by low solubility, incomplete pharmacokinetics, uncertain active tumour exposure, sparse repeat-dose safety data, and the absence of direct comparisons between free CA and candidate formulations [5,6,10,14,37]. The main question is whether CA can be administered at a tolerable dose that achieves sufficient and reproducible tumour exposure for sustained antitumour benefit.

### 1.3. Aim, Evaluation Framework, and Scope of This Review

Research on CA spans phytochemistry, liver cancer cell phenotypes, molecular regulation, tumour suppression in animals, mechanisms in other disease models, and formulation development. Yet the models, interventions, and evidential strength differ substantially across studies [3,4,5,6,7,10,12,14]. Accordingly, this review defines HCC as its primary focus and organises the literature within a tiered evidence framework.

Effects of isolated CA on proliferation, cell-cycle regulation, cell death, migration, and tumour growth in HCC cells and liver tumour models are treated as direct evidence. The causal strength of each proposed mechanism is then evaluated according to the supporting evidence, including molecular changes, functional perturbation, and direct binding [3,4,5,6,7,12,13]. By contrast, mechanistic findings from non-HCC tumour types and from metabolic, inflammatory, immune, and fibrotic models are treated as supportive evidence. They identify candidate regulatory nodes but do not establish those mechanisms in HCC [8,10,11]. Translational feasibility is evaluated through seven domains: physicochemical properties, gastrointestinal absorption, metabolism, safety, structural modification, delivery systems, and combination treatment. Within this analysis, a distinction is made among free CA, CA-encapsulating formulations, and delivery systems that use CA itself as a functional material [10,11,14,37,38,39,40].

The review first outlines its search and appraisal methods and then examines the chemical and pharmaceutical basis of CA, direct anti-HCC evidence, cross-model mechanisms, and translational barriers. This organisation separates directly supported effects, untested hypotheses, and current constraints on therapeutic development. CA is therefore positioned as a natural lead with preclinical anti-HCC support but without a complete candidate-level evidence chain [10,11,14]. The evidence-stratified framework adopted in this review is summarised in Figure 1.

## 2. Literature Search and Evidence Appraisal

We conducted a critical narrative review based on structured searches of PubMed, Scopus, and the Web of Science Core Collection from database inception to 6 May 2026. To identify evidence directly related to hepatocellular carcinoma, we first combined terms for corosolic acid (“corosolic acid”, “corosolic”, “colosolic acid”, “2α-hydroxyursolic acid”, and “4547-24-4”) with terms for HCC (“hepatocellular carcinoma”, “hepatocellular cancer”, “liver cancer”, “liver carcinoma”, “hepatoma*”, “hepatocarcinoma*”, and “HCC”). Because direct HCC studies were limited, the search was broadened by combining the corosolic-acid terms with cancer- and mechanism-related terms covering proliferation, apoptosis, cell-cycle regulation, migration, invasion, angiogenesis, ferroptosis, autophagy, oxidative stress, and selected signalling pathways. The complete Boolean search strings are provided in Supplementary Table S1. Reference lists of relevant primary studies and reviews were also screened, and targeted searches were conducted for physicochemical properties, pharmacokinetics, formulation, botanical sources, and structural modification.

Original studies were considered direct HCC evidence when they examined isolated CA or a clearly defined CA preparation in liver cancer cells or liver tumour models and reported at least one relevant biological outcome, such as proliferation, cell-cycle regulation, cell death, migration, tumour growth, or treatment response. Studies were excluded from this evidence set when they did not test CA itself, examined a botanical mixture in which the effect of CA could not be distinguished, lacked a liver tumour model, or did not report an HCC-relevant outcome. Studies using HepG2 or SK-Hep1 cells were retained when relevant, but the limitations of these models were considered explicitly rather than treating them as equivalent to classic HCC cell lines.

Non-HCC studies served a different purpose and were included only when they examined a mechanism already implicated by direct HCC evidence, addressed an HCC-relevant microenvironmental process such as angiogenesis, myeloid immunosuppression, or fibrosis, or provided pharmacokinetic, safety, formulation, or structural information relevant to CA development. To avoid selecting non-HCC studies merely because they reported anticancer activity, we retained only studies that linked a relevant biological effect to molecular or pathway-level evidence. These studies were therefore treated as supportive evidence for HCC-relevant hypotheses rather than as direct HCC evidence.

For both evidence groups, only English-language publications were considered. Reviews were used primarily to provide background and identify additional relevant studies and were not treated as primary mechanistic evidence. Because the included studies differed substantially in experimental models, treatments, and outcomes, their findings were compared and synthesised narratively. The complete database-specific search strategies, search combinations, and retrieval results are provided in Supplementary Tables S1 and S2.

## 3. Chemical Structure, Plant Sources, and Physicochemical Properties of Corosolic Acid

CA is an ursane-type pentacyclic triterpenoid with the molecular formula C_30_H_48_O_4_ and a molar mass of approximately 472.7 g mol^−1^ [9,14]. It shares an ursane scaffold with natural pentacyclic triterpenoids such as ursolic acid (UA) and asiatic acid. While CA differs from UA by an additional 2α-hydroxyl group, it is described as 2α-hydroxyursolic acid [9,11,14,15]. The structural relationships between CA and related pentacyclic triterpenoids are illustrated in Figure 2.

CA was first isolated from *Lagerstroemia speciosa* leaves and was later found in *Crataegus pinnatifida* var. *psilosa* [9,41,42,43]. Other reported sources include *Eriobotrya japonica*, *Orthosiphon stamineus*, *Psidium guajava*, *Prunella vulgaris*, *Perilla frutescens* var. *japonica*, *Saurauia roxburghii*, *Actinidia valvata*, and *Salvia syriaca* [9,17,44,45,46,47,48]. Because existing records are fragmented, the botanical distribution of CA has not been systematically assessed using consistent taxonomic and analytical criteria [9,17,41,42,44,45,46,49,50].

Quantitative studies suggest that CA content varies with plant species, medicinal part, geographical origin, and processing [44,51]. Guava leaves contained nearly 1% CA, whereas none was detected in the fruit. Hydrochloric acid hydrolysis increased the measured leaf content to 1.54%, more than 48.6% above the untreated value [44]. The increase after hydrolysis suggests that some CA may occur in conjugated forms. It also indicates that sample preparation can influence the measured CA content. Ultra-performance liquid chromatography–mass spectrometry (UPLC–MS) analysis of cranberry materials and processed products showed that pentacyclic triterpenoid profiles, including CA, varied with source and processing [51]. Differences in sampled tissue, extraction, hydrolysis, and detection currently prevent firm conclusions about reproducible interspecies differences in CA content [44,51].

Chromatographic methods have been used to quantify CA alongside related triterpenoids in *Prunella vulgaris* and processed botanical materials [46,51]. UPLC–MS enables the simultaneous profiling of CA and related triterpenoids. Hydroxypropyl-β-cyclodextrin (HP-β-CD) has also been reported to improve the analytical separation of CA from its structural isomer, maslinic acid [52]. However, standardised procedures for sample preparation, calibration, and method validation are still required for reliable cross-study comparisons.

The rigid pentacyclic scaffold and pronounced hydrophobicity of CA confer low aqueous solubility, which may constrain dissolution, formulation, and oral bioavailability [10,11,14]. A qualitative experimental study reported that purified CA was insoluble in water but soluble in dimethyl sulfoxide, *N*,*N*-dimethylformamide, tetrahydrofuran, and ethanol [53]. However, that study did not determine equilibrium solubility. No standardised experimental values for aqueous solubility, the octanol/water partition coefficient (logP), the pH-dependent distribution coefficient (logD), or the acid dissociation constant (pKa) were identified. Available database values are model-based. The Human Metabolome Database (HMDB) lists predicted logP values of approximately 5.49–5.51, a predicted logarithm of molar solubility (logS) of −5.01, a corresponding predicted aqueous solubility of approximately 4.57 mg/L, and a predicted strongest acidic pKa of approximately 4.74 [54]. A reliable logD value at a specified pH was not identified. Other predicted descriptors include a physiological charge of −1, a polar surface area of 77.76 Å^2^, three hydrogen-bond donors, four hydrogen-bond acceptors, and one rotatable bond [54]. These estimates are consistent with high lipophilicity and low water solubility but should not be interpreted as experimental measurements.

Poor aqueous solubility does not preclude measurable gastrointestinal absorption [37]. In situ rat studies reported gastric absorption of approximately 20–40% and absorption across the examined intestinal segments. Absorption rate constants were broadly similar across intestinal regions, whereas effective permeability varied by site and concentration [37]. Several putative metabolites were also detected in plasma and bile, and cytochrome P450 1A2 (CYP1A2) and cytochrome P450 3A4 (CYP3A4) were implicated in CA metabolism [37]. However, these studies did not determine absolute oral bioavailability or establish whether sufficient systemic or tumour exposure can be achieved.

CA has also been reported to form a 1:1 inclusion complex with HP-β-CD, with stronger binding than that observed for maslinic acid [52]. This finding supports the feasibility of cyclodextrin inclusion but does not demonstrate improved CA exposure in vivo. Broader formulation strategies for poorly soluble phytochemicals are discussed in Section 6.2.

## 4. Direct Effects and Mechanisms of Corosolic Acid in Hepatocellular Carcinoma

### 4.1. Direct Anti-HCC Evidence: From Activity In Vitro to Validation In Vivo

Available evidence for the direct anti-HCC effects of CA remains preclinical, with no demonstrated clinical benefit in patients. Early screening studies showed that isolated or biotransformed CA inhibited HepG2 cell growth [15,17,18], whereas subsequent studies extended the assessed endpoints to proliferation, cell-cycle progression, apoptosis, migration, ferroptosis susceptibility, and tumour growth in vivo [3,4,5,6,7,12,13,16]. Although these findings support direct activity against HCC, the studies remain few and heterogeneous in the model, dose, exposure time, and endpoint. CA should therefore be described as a lead compound with preclinical anti-HCC activity rather than an established treatment.

Model identity limits part of this evidence. HepG2 is hepatoblastoma-derived rather than a conventional HCC cell line [55], whereas SK-HEP-1 has endothelial characteristics and lacks key hepatocyte properties [56]. Findings from these models are retained because they form part of the CA literature, but they should not be treated as equivalent to results from authenticated HCC cell lines. The direct preclinical evidence, experimental conditions, and principal limitations are summarised in Table 1.

### 4.2. Inhibition of HCC Cell Proliferation and Cell-Cycle Progression

Suppressed proliferation and colony formation are recurring effects of CA in liver cancer cells. In Bel-7404 and HepG2 cells, CA produced dose-dependent inhibition and increased the gap 2/mitosis (G2/M) population [13]. Because the remaining direct evidence does not establish the same phase of arrest across multiple HCC models, CA should not be characterised broadly as a compound that invariably induces G2/M arrest. The cell-cycle phenotype is better reported in relation to the specific model and treatment conditions.

Proteomic and database analyses from the same study identified prolyl 4-hydroxylase subunit alpha 2 (P4HA2) as a candidate regulator associated with CA activity [13]. Public datasets showed increased P4HA2 expression in HCC tissue and an association with poor prognosis, while CA reduced P4HA2 protein together with proliferation and colony formation. However, the study did not test whether P4HA2 knockdown, overexpression, or rescue altered the response to CA. P4HA2 should therefore be treated as an associated candidate regulator rather than a validated causal mediator or direct target [13].

Yes-associated protein (YAP) is supported by more extensive functional evidence [6]. CA reduced YAP stability, increased its ubiquitination, shifted YAP from the nucleus to the cytoplasm, and reduced its interactions with the transcriptional regulators cAMP response element-binding protein, TEA domain transcription factor, and runt-related transcription factor 2. CA also increased the interaction between YAP and the E3 ubiquitin ligase mouse double minute 2 homolog (MDM2). In xenografts, MDM2 knockdown or YAP overexpression weakened tumour suppression by CA, supporting a functional contribution of MDM2-dependent YAP inactivation in vivo. Direct binding of CA to YAP or MDM2 has not been demonstrated, so this pathway is better viewed as a downstream regulatory network [6].

Under high-glucose conditions, CA was further linked to cyclin-dependent kinase 19 (CDK19)–YAP–O-linked β-N-acetylglucosamine (O-GlcNAc) regulation [7]. High glucose increased proliferation and colony formation, together with CDK19, YAP, O-GlcNAc transferase (OGT), and global O-GlcNAcylation. CA lowered CDK19 and reversed these associated changes, whereas CDK19 overexpression partly restored YAP and O-GlcNAcylation after CA treatment. These results indicate that CDK19 contributes functionally to the response and suggest that CA restricts the proliferative advantage conferred by high glucose. They do not, however, establish CA as a direct CDK19 inhibitor [7].

The relationship between YAP abundance and CA activity may not be unidirectional. A separate study found that CA increased large tumour suppressor kinase 1 (LATS1)-mediated YAP phosphorylation and beta-transducin repeat-containing protein (βTrCP)-dependent degradation, but excessive YAP loss appeared to limit CA-induced apoptosis [19]. Actinomycin D increased YAP abundance, reduced βTrCP, enhanced apoptosis, and lowered the half-maximal inhibitory concentration of CA. This result differs from a simple model in which lower YAP necessarily strengthens CA activity, indicating that the role of YAP may depend on the cellular state, endpoint, and combination conditions [6,19]. Because evidence for the actinomycin D combination is mainly in vitro, its efficacy and safety in vivo remain unknown.

Thus, P4HA2, MDM2–YAP, and CDK19–YAP–O-GlcNAcylation represent separate regulatory explanations for the antiproliferative effects of CA rather than components of one established pathway [6,7,13,19]. Functional perturbation supports the involvement of MDM2–YAP and CDK19 more strongly than P4HA2. At present, these studies identify pathway dependence and downstream regulation but not a common direct molecular target of CA.

### 4.3. Cell Death: Mitochondrial Apoptosis, Endoplasmic Reticulum Stress, and Ferroptosis

CA affects several death processes in liver cancer cells. Mitochondrial apoptosis has the most consistent support, endoplasmic reticulum (ER) stress may contribute upstream, and HERPUD1-associated glutathione depletion mainly sensitises cells to ferroptosis [3,6,12,16]. Lipid peroxidation alone does not establish ferroptosis [20].

#### 4.3.1. Mitochondrial Dysfunction-Mediated Apoptosis

In HepG2 cells, CA inhibited growth and induced apoptosis in a dose-dependent manner, with a half-maximal inhibitory concentration (IC50) of approximately 40 μM [16]. Treatment increased intracellular reactive oxygen species (ROS), reduced mitochondrial membrane potential, and promoted cytochrome c redistribution from mitochondria to the cytosol. These changes are consistent with altered mitochondrial outer-membrane permeability. Caspase-3 and caspase-9 cleavage was also observed across several liver cancer cell lines [6], connecting mitochondrial dysfunction with execution of the intrinsic apoptotic programme. The recurrence of mitochondrial depolarisation, cytochrome c release, and caspase cleavage across the available studies provides stronger support than a single molecular marker [6,16]. Nevertheless, pathway-specific genetic rescue remains limited, and no individual mitochondrial component has been shown to be indispensable for the response.

#### 4.3.2. Link Between Endoplasmic Reticulum Stress and Mitochondrial Apoptosis

Endoplasmic reticulum stress may act upstream of mitochondrial apoptosis induced by CA [3]. Transcriptomic analysis and cellular validation showed activation of PERK–eIF2α–ATF4 signalling together with mitochondrial apoptotic changes. Salubrinal pretreatment markedly attenuated CA-induced apoptosis, while CCAAT/enhancer-binding protein homologous protein (CHOP) knockdown weakened both endoplasmic-reticulum-stress proteins and apoptotic phenotypes. These interventions indicate that endoplasmic reticulum stress contributes functionally rather than merely accompanying CA exposure [3].

On this basis, CA may disrupt endoplasmic reticulum proteostasis and activate the PERK–eIF2α–ATF4–CHOP response, which is then relayed to the mitochondrial apoptotic programme [3]. The use of pharmacological attenuation and CHOP knockdown gives this model more weight than expression changes alone. However, the connection between individual ER-stress nodes and mitochondrial permeabilisation remains incompletely defined, and this mechanistic interpretation is based on a single study without independent replication in other HCC models or validation in vivo [3]. It should therefore be regarded as an intervention-supported working model rather than a universal pathway in HCC.

#### 4.3.3. Ferroptosis Susceptibility Mediated by the HERPUD1–GSH Axis

Beyond apoptosis, CA may weaken antioxidant defences and increase the susceptibility of liver cancer cells to ferroptosis [12]. Ferroptosis is characterised by iron-dependent accumulation of lipid peroxides, while glutathione (GSH) helps maintain redox balance and restrict lipid peroxidation. In the reported sensitisation experiments, cells were pretreated with 10 μM CA for 1 h before exposure to erastin or RSL3. CA alone at this concentration caused limited cell death, whereas the combinations increased lipid ROS accumulation and cell death. Ferrostatin-1 attenuated the combination effects, supporting CA as a ferroptosis sensitiser rather than an independent, canonical ferroptosis inducer [12].

Mechanistically, CA upregulated homocysteine-inducible endoplasmic reticulum stress protein with ubiquitin-like domain 1 (HERPUD1), which stabilised MDM2 and increased the ubiquitination of glutathione synthetase (GSS) [12]. The resulting decrease in GSS protein, glutathione synthesis, and intracellular GSH weakened the antioxidant capacity and favoured lipid peroxide accumulation. HERPUD1 loss attenuated the effects of CA on GSH metabolism and ferroptosis susceptibility, providing genetic support for this mechanism. In Bel-7402 xenografts, combination treatment with CA and the ferroptosis inducer imidazole ketone erastin (IKE) produced greater tumour suppression, whereas HERPUD1 loss weakened the response [12]. The evidence therefore supports HERPUD1-dependent sensitisation in vivo but does not show that CA alone induces a complete ferroptotic process.

A separate study reported lipid-peroxidation-associated non-apoptotic death in SK-HEP-1 and Huh7 cells [20]. α-Tocopherol reduced both lipid peroxidation and cell death, whereas a pan-caspase inhibitor and inhibitors of necroptosis or ferroptosis, including ferrostatin-1 and deferoxamine (DFO), did not block the process. This phenotype should therefore be distinguished from the HERPUD1-associated ferroptosis-sensitising effect [12,20]. Overall, mitochondrial apoptosis has the most consistent support, whereas the ferroptosis evidence primarily concerns sensitisation to another inducer. Current evidence does not establish that organelle stress, apoptosis, and ferroptosis susceptibility form one continuous death programme shared by all HCC models. The distinct evidence branches are summarised in Figure 3.

### 4.4. Inhibition of Liver Cancer Cell Migration: VEGFR2–Src–FAK–Cdc42 Signalling

Migration is considered here as an invasion-relevant phenotype rather than evidence of metastasis. In Huh7 cells, the half-maximal concentration for migration inhibition was approximately 2.5 μM, compared with a cytotoxic concentration of approximately 50 μM [5]. This difference indicates that migration was suppressed below overtly cytotoxic concentrations and was unlikely to result entirely from cell death or reduced proliferation.

Mechanistically, CA inhibited purified VEGFR2 kinase activity in an adenosine diphosphate (ADP)-Glo assay, with a reported IC50 of 0.95 μM [5]. In Huh7 cells, CA reduced VEGFR2 tyrosine phosphorylation by approximately 70% without a comparable change in total VEGFR2. Small interfering RNA (siRNA)-mediated VEGFR2 knockdown also attenuated CA-induced migration inhibition, supporting the involvement of VEGFR2 in this phenotype. Consistent with this interpretation, CA reduced Src and FAK phosphorylation, active Cdc42, and the filamentous actin/globular actin (F-actin/G-actin) ratio [5]. Molecular docking predicted that CA could occupy the adenosine triphosphate (ATP)-binding pocket of VEGFR2 and interact with residues including Glu883. However, docking does not demonstrate physical binding, and the study did not include a direct binding or cellular target-engagement assay. Thus, the evidence supports VEGFR2 kinase inhibition and the involvement of a VEGFR2-associated migratory pathway but is insufficient to define VEGFR2 as a confirmed direct molecular target of CA [5].

In subcutaneous Huh7 xenografts, intraperitoneal CA at 5 mg/kg/day for 21 days reduced tumour volume and weight and lowered VEGFR2 and FAK phosphorylation in tumour tissue [5]. These findings support modulation of the pathway in vivo, but the model assessed tumour growth rather than local invasion or distant metastasis. The mechanism is also based mainly on one study. CA should therefore be described as inhibiting HCC cell migration through a VEGFR2-associated pathway, not as having an established anti-metastatic effect.

### 4.5. Tumour Suppression In Vivo and the Limits of the Evidence

Antitumour activity has been reported in conventional subcutaneous xenografts, a streptozotocin (STZ)-hyperglycaemic Bel-7404 xenograft, combination studies with sorafenib or a ferroptosis inducer, and a Walker-256 orthotopic liver-tumour implantation model combined with associating liver partition and portal vein ligation for staged hepatectomy (ALPPS) [4,5,6,7,12]. Recurrence across these models supports in vivo activity, but differences in tumour cells, hosts, metabolic context, dosing, and endpoints preclude a single mechanistic explanation. The animal evidence is therefore most informative when each model is interpreted according to the question it addresses.

In subcutaneous Huh7 xenografts, intraperitoneal CA at 5 mg/kg/day for 21 days reduced tumour volume and weight and enhanced the inhibitory effect of a lower dose of sorafenib [5]. The absence of an obvious body-weight decrease suggests that overt systemic toxicity was not detected during this experiment. It cannot replace haematological, biochemical, or histopathological safety assessment. Similarly, improved tumour control with CA–IKE supports combination activity but does not establish the tolerability of sustained ferroptosis sensitisation [12].

The strongest mechanistic animal studies used genetic perturbation. In the MDM2–YAP study, CA increased the interaction between YAP and MDM2, promoted YAP ubiquitination, and reduced nuclear YAP. In xenografts, MDM2 knockdown or YAP overexpression weakened tumour suppression [6]. In the HERPUD1 study, CA upregulated HERPUD1, stabilised MDM2, increased GSS ubiquitination, and reduced GSH. HERPUD1 loss attenuated the effects of CA on GSH metabolism and weakened the CA–IKE response [12]. Together, these perturbation experiments support a functional role for MDM2-dependent YAP inactivation in CA-mediated tumour suppression and for HERPUD1-dependent GSH depletion in sensitisation to IKE. However, both studies relied on pre-engineered tumour cells implanted subcutaneously in immunodeficient hosts and did not reproduce spontaneous clonal heterogeneity or the full liver tumour microenvironment.

The hyperglycaemia model introduced host metabolism as an additional variable [7]. In STZ-treated mice bearing Bel-7404 subcutaneous xenografts, intratumoural CA at 10 mg/kg/day for 20 days lowered blood glucose, tumour burden, and CDK19–YAP–O-GlcNAc-associated markers [7]. Because glucose reduction may itself alter nutrient availability and tumour adaptation, the relative contributions of direct tumour cell activity and metabolic improvement remain unresolved. By contrast, the ALPPS-associated study used Walker-256 tumour tissue implanted into the middle liver lobe of male Sprague–Dawley rats, with CA administered intraperitoneally at 20 mg/kg every other day from postoperative day 3 to day 15 [4]. This is an orthotopic liver-tumour implantation model, not a standard HCC-cell-derived model. CA reduced tumour weight without clearly impairing regeneration and was accompanied by changes in macrophage markers, cluster of differentiation 8 (CD8)-positive cells, checkpoint markers, and vascular markers. These findings provide preclinical proof of concept for tumour growth suppression but do not establish clinically achievable tumour exposure, long-term benefit, efficacy in HCC arising in chronically diseased liver, or an adequate safety margin. They also do not identify a dominant mechanism in vivo.

Across the animal studies, the principal endpoints were short-term tumour volume, tumour mass, and tissue biomarkers [4,5,6,7,12]. Most models were subcutaneous xenografts, some studies used intraperitoneal administration, and oral tumour exposure was not established. The subcutaneous setting is useful for tumour growth and rescue experiments but does not reproduce liver-specific vascular, fibrotic, metabolic, and immune conditions. Pharmacokinetic–pharmacodynamic relationships, long-term survival, recurrence, metastasis, and systematic toxicity assessment were generally absent. Apart from the ALPPS-associated study, CA has been poorly tested in immunocompetent orthotopic HCC models with chronic underlying liver disease.

Taken together, CA suppresses tumour growth in several preclinical HCC models, with different degrees of functional support for MDM2–YAP, CDK19–YAP–O-GlcNAcylation, HERPUD1–GSH, and VEGFR2–FAK regulation [4,5,6,7,12]. These findings neither predict clinical efficacy nor identify a dominant mechanism in vivo. They also leave several microenvironmental mechanisms untested directly in HCC; evidence from other disease or tumour models should therefore be used only to formulate hypotheses for subsequent validation.

## 5. HCC-Relevant Mechanistic Hypotheses Requiring Direct Validation

Direct HCC studies establish the effects of CA on proliferation, cell death, migration, and tumour growth [3,4,5,6,7,12,13,16]. Evidence from other tumours or non-malignant models cannot demonstrate anti-HCC efficacy. It is useful only when it identifies a candidate mechanism that converges with direct HCC findings or addresses a feature of the liver tumour microenvironment that has not yet been tested. On this basis, the most relevant cross-model evidence concerns stress-associated apoptosis, GSS/GSH regulation, VEGFR2/FAK signalling, and immunosuppressive myeloid cells.

### 5.1. Convergence with HCC Evidence

Endoplasmic reticulum stress is one of the clearest points of convergence. In castration-resistant prostate cancer cells, CA activated inositol-requiring enzyme 1 (IRE1)/apoptosis signal-regulating kinase 1 (ASK1)/c-Jun N-terminal kinase (JNK) and PERK/eIF2α/ATF4/CHOP signalling, while knockdown of IRE1, PERK, or CHOP partly reduced cytotoxicity [21]. This finding is consistent with PERK/eIF2α/ATF4 activation, salubrinal rescue, and CHOP knockdown in HCC cells [3]. Together, the studies support PERK/CHOP as a recurrent stress-death node. They do not establish a common upstream trigger or show that the pathway operates across all HCC subtypes.

Regulation of glutathione metabolism provides a second point of convergence. In lung cancer cells, CA reduced YAP, GSS, and GSH and increased lipid ROS, linking ferroptosis-associated changes to reduced EMT-related phenotypes [22]. Direct HCC evidence instead connects GSS/GSH depletion to the HERPUD1/MDM2 axis and supports sensitisation to ferroptosis inducers [12]. The shared downstream change is loss of GSS/GSH, but the proposed upstream regulators differ. Therefore, a single YAP/HERPUD1/MDM2/GSS pathway should not be inferred without epistasis and rescue experiments in HCC.

Vascular studies also converge partly with the VEGFR2/Src/FAK/Cdc42 mechanism reported in Huh7 cells [5]. CA inhibited endothelial and lymphatic–endothelial proliferation, migration, and tube formation and reduced blood- and lymphatic-vessel density in a CT-26 tumour model, accompanied by lower FAK and extracellular signal-regulated kinase signalling [23]. These findings increase the plausibility of vascular effects but do not show that CA inhibits angiogenesis or lymphangiogenesis in HCC. Hepatic endothelial co-cultures and orthotopic models are required to separate effects on tumour cell migration from effects on tumour vasculature.

### 5.2. Myeloid Immunity and Fibrosis

Evidence linking CA to STAT3-dependent myeloid regulation is relevant to the immune changes observed in the ALPPS-associated HCC model [4]. Across macrophage, glioblastoma, ovarian cancer, and murine sarcoma models, CA reduced STAT3 activation and weakened cluster of differentiation 163 (CD163)-positive macrophage, tumour-associated macrophage, or myeloid-derived suppressor cell functions [24,25,26,27]. Reduced interleukin-10 production, weaker suppression of T-cell activation, and increased lymphocyte infiltration support an effect on immunosuppressive myeloid cells. Targeted CA liposomes also affected CD163-positive macrophages, but their cellular selectivity arose from antibody-mediated delivery rather than free CA [38]. These studies used different tumour types and intervention forms, and the HCC model did not include STAT3 perturbation, macrophage depletion, or CD8 cell blockade. The evidence therefore supports a candidate immune mechanism rather than immune causality in HCC.

The fibrotic background is another HCC-relevant context. In steatohepatitis and hepatic stellate cell models, CA reduced transforming growth factor beta 1 (TGF-β1)/SMAD family member 2 (SMAD2) signalling, inflammation, collagen-associated markers, and fibrosis [28]. The orthotopic HCC study also reported lower tumour transforming growth factor beta (TGF-β) and changes in macrophage and vascular markers [4]. These findings suggest that CA may modify the diseased liver environment, but antifibrotic activity cannot be equated with regression of an established tumour. This hypothesis requires immunocompetent HCC models with underlying fibrosis and separate assessment of tumour cell and stromal responses.

### 5.3. Limits and Validation Priorities

Cross-model consistency should not be interpreted as a complete anti-HCC mechanism. The same pathway may produce different outcomes according to cell type and disease context. For example, AMPK, mTOR, and autophagy changes were associated with tumour cell death or chemosensitisation in gastric cancer but with cytoprotection in hepatocytes, cardiomyocytes, and chondrocytes [29,30,31,32,33,34]. These findings do not justify adding AMPK or autophagy to the established HCC mechanism. They instead indicate that pathway direction, autophagic flux, clonogenic survival, and normal-hepatocyte toxicity must be measured together in HCC models.

Accordingly, cross-model evidence should be used to prioritise experiments rather than extend conclusions. PERK/CHOP, GSS/GSH, VEGFR2/FAK, and STAT3-associated myeloid regulation have the closest links to current HCC findings, but each still requires pathway-specific perturbation and phenotypic rescue. Validation should proceed in HCC cell lines, organoids, hepatic stromal co-cultures, and immunocompetent orthotopic models. Table 2 summarises the cross-model mechanisms, current evidence levels, and priorities for HCC-specific validation.

## 6. Translational Barriers and Development Priorities

CA has progressed from in vitro activity to tumour suppression in xenograft and orthotopic HCC models. However, evidence on active exposure, formulation performance, combination dosing, and systematic safety remains disconnected [4,5,6,37]. Translation therefore depends less on adding further pathway observations than on linking dose, tumour exposure, efficacy, and toxicity in HCC-relevant models.

### 6.1. Exposure, Pharmacokinetics, and Safety

The rigid pentacyclic scaffold and hydrophobicity of CA result in low aqueous solubility, which may limit dissolution and oral formulation performance [10,11,14,45]. Rat gastric absorption and intestinal perfusion experiments nevertheless showed measurable gastrointestinal absorption. Gastric absorption was approximately 20–40%, and absorption occurred throughout the examined intestinal segments [37]. These findings do not establish adequate systemic or tumour exposure. The central question is whether an acceptable oral dose can sustain active concentrations in non-tumour liver and HCC tissue.

This question cannot be answered from the current efficacy studies. HCC cell activity near 40 μM and tumour suppression at 5 mg/kg/day have not been connected by plasma, liver, or tumour concentrations [5,6]. Five putative metabolites were detected in rat plasma and bile, while microsomal experiments implicated CYP1A2 and CYP3A4 in CA metabolism [37]. The activity and toxicity of the parent compound and individual metabolites remain unresolved. Future studies should quantify total and unbound CA and major metabolites after oral and parenteral administration and relate these measurements to tumour response.

Available studies have not identified a prominent acute toxicity signal, but they cannot define an HCC-relevant safety margin. Reviews of CA-containing materials and animal studies in other disease models reported no clear severe toxicity, including studies using relatively high doses [35,36,43]. Differences in test material, dose, disease model, and observation period prevent direct comparison. Purified CA and selected formulations require repeat-dose toxicity, toxicokinetic, biochemical, and histopathological assessment. Because many patients with HCC have chronic liver disease, tolerance should be examined in both healthy and fibrotic or steatohepatitic livers.

### 6.2. Formulation and Combination Development

Most direct HCC studies have tested free CA, and no HCC study has directly compared free CA with an optimised formulation [4,5,6]. Existing delivery systems must also be distinguished by the role of CA. In CA-loaded formulations, CA is the therapeutic payload, as in prolamin nanoparticles or anti-CD163 liposomes [38,40]. In CA-functionalised carriers, CA or its derivatives form part of the delivery material and may alter uptake, penetration or immune responses while another agent remains the principal payload [39,57,58,59]. Cyclodextrin nanosponges have been investigated as bioenhancers for poorly soluble phytochemicals by increasing apparent solubility and modulating compound release [60]. However, this evidence is not specific to CA and does not demonstrate improved CA exposure or efficacy. The results obtained with these systems do not demonstrate improved bioavailability or efficacy of CA monotherapy. Representative studies and their evidence boundaries are compared in Table 3.

An HCC formulation study should compare free CA, empty carrier, CA-free carrier, and the complete formulation. Release, unbound CA exposure, liver and tumour distribution, efficacy, and hepatic toxicity should be measured in the same experiment. A formulation advantage should be claimed only when increased tumour exposure or therapeutic index can be attributed to CA delivery rather than to the carrier or a co-loaded drug.

Structural modification may improve activity or distribution, but increased cytotoxicity alone is insufficient. CA-derived aromatic amides and mitochondria-targeted conjugates showed stronger activity in non-HCC tumour cells [61,62,63]. Some mitochondria-targeted compounds also impaired oxidative phosphorylation in normal liver mitochondria [62]. Derivatives should therefore be compared with parent CA for solubility, metabolic stability, HCC selectivity, normal-hepatocyte toxicity, exposure, and therapeutic window before further development.

Direct HCC combination evidence currently includes sorafenib, actinomycin D, and ferroptosis inducers [5,12,19]. These studies support greater tumour inhibition or cell death under selected conditions, but they do not establish clinically feasible schedules. Combination studies should use dose matrices and formal interaction models, compare concurrent and sequential treatment, and measure exposure to both agents. CA-functionalised chemotherapy carriers must be evaluated separately from free CA administered with chemotherapy.

### 6.3. HCC-Specific Validation Roadmap

Candidate selection should begin with CA of defined purity and formulations of reproducible composition. Studies should establish dose–exposure–response relationships while measuring parent CA, metabolites, efficacy, and toxicity in parallel. CYP1A2- and CYP3A4-mediated metabolism and potential drug interactions also require direct evaluation [37]. These experiments should identify whether oral dosing reaches tumour exposure associated with the effects observed in vitro and after parenteral administration.

Model progression should move from two-dimensional screening to HCC organoids or patient-derived systems and then to orthotopic, immunocompetent models with fibrosis or steatohepatitis. The same programme should assess tumour burden, survival, invasion or metastasis, immune context, and non-tumour liver injury or regeneration [4,5,6]. Mechanistic rescue experiments should be incorporated into these models rather than conducted separately from pharmacokinetic and safety studies.

Reproducible manufacture is also required. Natural-source CA needs defined botanical origin, purification, purity, impurity profile, and batch consistency. Derivatives require controlled synthesis and impurity assessment, while nanoformulations additionally require defined loading, particle size, release, storage stability, and scale-up [10,14,45]. Before clinical testing, a candidate should provide stable active tumour exposure, reproducible benefit over free CA or an appropriate control, and an adequate safety margin. At present, CA remains a preclinical lead because this candidate-level evidence chain and human anti-HCC evidence are absent.

## 7. Conclusions

This review integrates the available preclinical evidence on CA in HCC by distinguishing direct HCC findings from cross-model mechanistic hypotheses and translational evidence. This framework clarifies which observations support antitumour activity, which remain hypothesis-generating, and where extrapolation beyond HCC-specific evidence becomes uncertain.

Preclinical studies indicate that CA can affect tumour growth, cell death, and migration in experimental HCC models, with several effects also observed in vivo. However, mechanistic support remains uneven. Some pathways are supported by functional perturbation, whereas others rely mainly on molecular associations or evidence from non-HCC models. CA should therefore be regarded as a preclinical natural-product candidate rather than a mechanistically established or clinically validated HCC therapy.

Cross-model findings on metabolism and autophagy, inflammatory and immune signalling, and fibrotic tissue remodelling provide testable hypotheses for HCC-specific studies. The main unresolved gaps are the absence of a defined dose–exposure–response relationship, limited validation in disease-relevant HCC models, insufficient systemic safety data, and the absence of matched HCC studies directly comparing free CA with candidate formulations. Together, these gaps define the evidence needed to judge whether CA warrants further development.

## Figures and Tables

**Figure 1:**
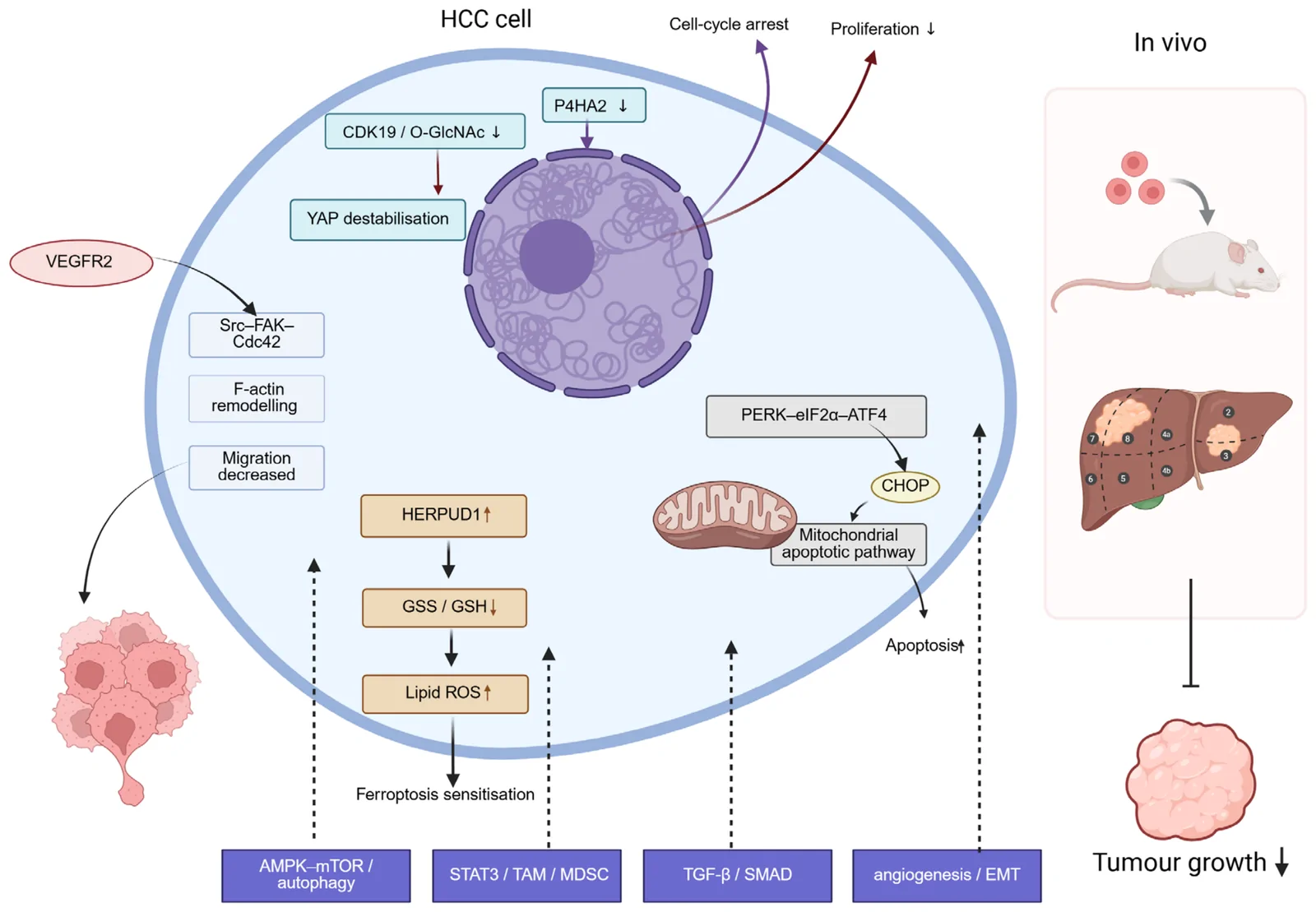
Evidence-stratified overview of the preclinical effects and proposed mechanisms of corosolic acid in hepatocellular carcinoma. Solid arrows indicate regulatory relationships investigated directly in HCC models, whereas dashed arrows from the purple boxes indicate hypotheses derived from other tumour or disease models that require direct validation in HCC. Direct HCC evidence supports VEGFR2–Src–FAK–Cdc42-associated migration inhibition; CDK19/O-GlcNAc–YAP- and P4HA2-associated growth control; PERK–eIF2α–ATF4–CHOP-associated apoptosis; and HERPUD1–GSS/GSH-associated ferroptosis sensitisation. The right-hand panel summarises tumour growth suppression observed in animal models. AMPK–mTOR–autophagy, STAT3-associated myeloid regulation, TGF-β/SMAD-associated stromal remodelling, and angiogenesis/EMT remain cross-model hypotheses. These pathways are presented as separate evidence-supported modules and should not be interpreted as a single linear mechanism. ↑ and ↓ indicate increases and decreases, respectively. The labels 2, 3, 4a, 4b, and 5–8 on the liver denote Couinaud liver segments. Abbreviations: AMPK, adenosine monophosphate-activated protein kinase; ATF4, activating transcription factor 4; CA, corosolic acid; Cdc42, cell division cycle 42; CDK19, cyclin-dependent kinase 19; CHOP, CCAAT/enhancer-binding protein homologous protein; eIF2α, eukaryotic translation initiation factor 2 alpha; EMT, epithelial–mesenchymal transition; F-actin, filamentous actin; FAK, focal adhesion kinase; GSH, glutathione; GSS, glutathione synthetase; HCC, hepatocellular carcinoma; HERPUD1, homocysteine-inducible endoplasmic reticulum stress protein with ubiquitin-like domain 1; MDSC, myeloid-derived suppressor cell; mTOR, mechanistic target of rapamycin; O-GlcNAc, O-linked β-N-acetylglucosamine; P4HA2, prolyl 4-hydroxylase subunit alpha 2; PERK, protein kinase R-like endoplasmic reticulum kinase; ROS, reactive oxygen species; SMAD, small mothers against decapentaplegic; Src, proto-oncogene tyrosine-protein kinase Src; STAT3, signal transducer and activator of transcription 3; TAM, tumour-associated macrophage; TGF-β, transforming growth factor beta; VEGFR2, vascular endothelial growth factor receptor 2; YAP, Yes-associated protein. Created with BioRender.com.

**Figure 2:**
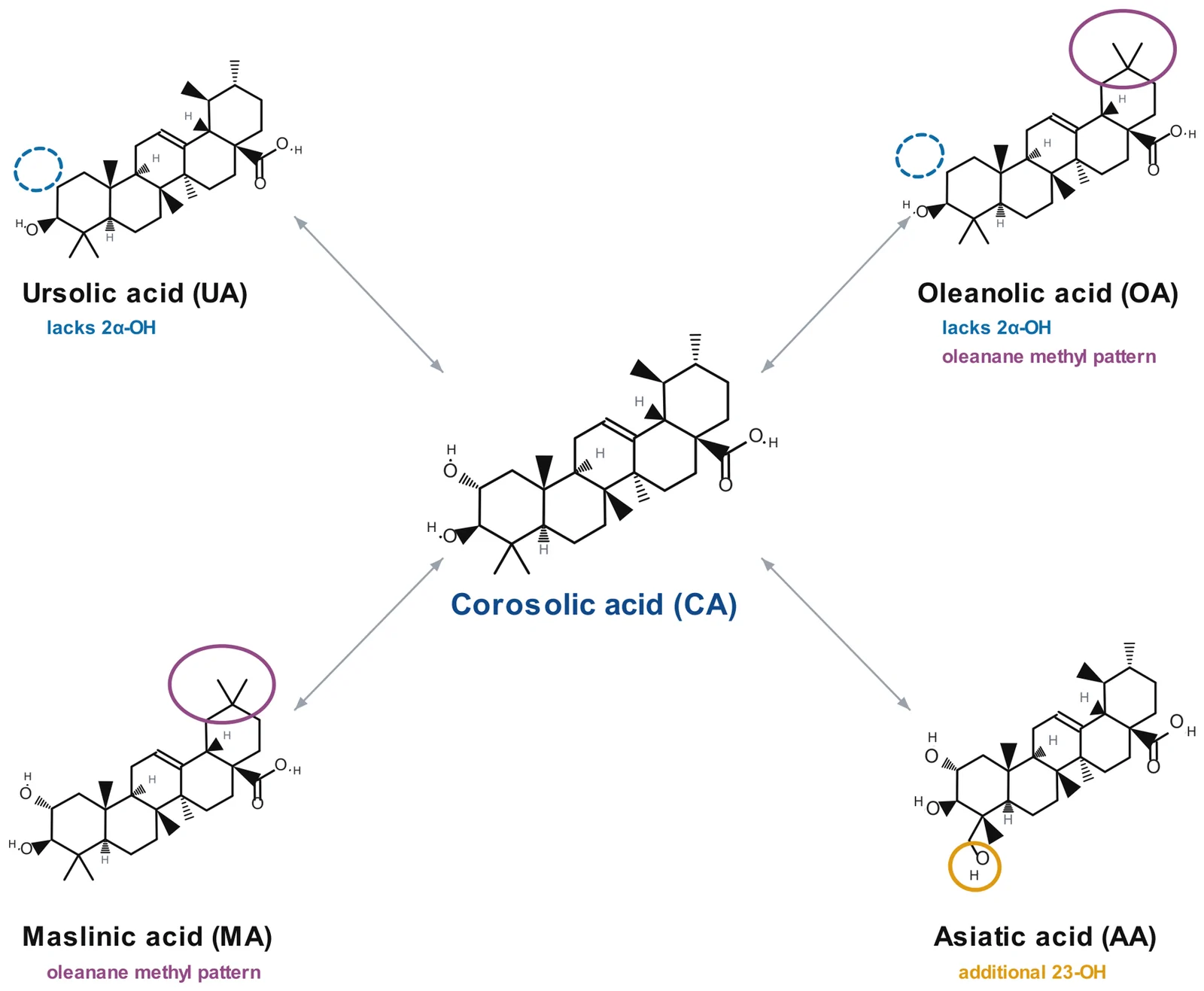
Structural relationships between corosolic acid and selected pentacyclic triterpenoids. Corosolic acid (CA) is shown as the reference structure. Compared with CA, ursolic acid (UA) lacks the 2α-hydroxyl group; oleanolic acid (OA) lacks the 2α-hydroxyl group and has the oleanane rather than ursane methyl-substitution pattern around C-19/C-20; maslinic acid (MA) retains the 2α,3β-dihydroxylation pattern but has the oleanane scaffold; and asiatic acid (AA) contains an additional 23-hydroxyl group. Blue dashed markers indicate a group absent relative to CA, orange highlights indicate an additional substituent, and purple outlines indicate the ursane–oleanane scaffold difference. Double-headed connectors denote structural comparisons rather than chemical or biosynthetic conversions.

**Figure 3:**
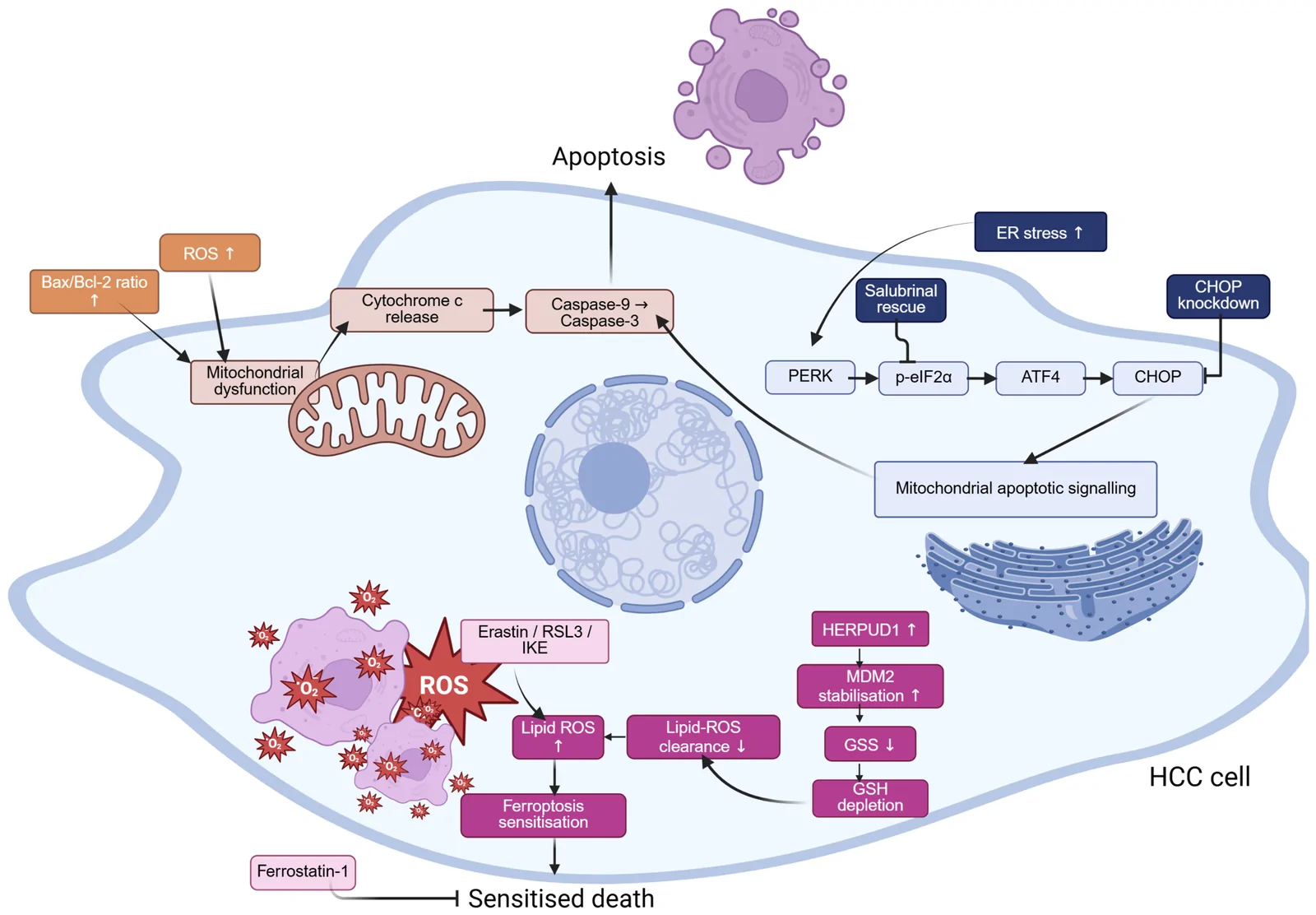
Evidence-supported cell-death pathways and ferroptosis sensitisation associated with corosolic acid in hepatocellular carcinoma models. Corosolic acid (CA) promotes mitochondrial apoptosis through an increased Bax/Bcl-2 ratio, reactive oxygen species (ROS) accumulation, mitochondrial dysfunction, cytochrome c release, and sequential caspase-9 and caspase-3 activation. The endoplasmic reticulum (ER) stress branch involves PERK–p-eIF2α–ATF4–CHOP signalling and converges on mitochondrial apoptotic signalling. Attenuation by salubrinal and CHOP knockdown provides functional support for this route. In the ferroptosis-sensitisation branch, CA-associated HERPUD1 upregulation stabilises MDM2, promotes GSS loss and GSH depletion, impairs lipid-ROS clearance, and enhances responses to erastin, RSL3, or IKE. The attenuation of these effects by ferrostatin-1 or HERPUD1 loss provides functional support for this mechanism. The available evidence therefore supports CA as a ferroptosis sensitiser rather than as an independent ferroptosis inducer. ↑ and ↓ indicate increases and decreases, respectively. Abbreviations: ATF4, activating transcription factor 4; Bax, Bcl-2-associated X protein; Bcl-2, B-cell lymphoma 2; CA, corosolic acid; CHOP, CCAAT/enhancer-binding protein homologous protein; ER, endoplasmic reticulum; GSH, glutathione; GSS, glutathione synthetase; HCC, hepatocellular carcinoma; HERPUD1, homocysteine-inducible endoplasmic reticulum stress protein with ubiquitin-like domain 1; IKE, imidazole ketone erastin; MDM2, mouse double minute 2 homolog; PERK, protein kinase R-like endoplasmic reticulum kinase; p-eIF2α, phosphorylated eukaryotic translation initiation factor 2 alpha; ROS, reactive oxygen species; RSL3, RAS-selective lethal 3. Created with BioRender.com.

**Table 1:** Direct preclinical evidence for corosolic acid in hepatocellular carcinoma.

Evidence Domain	Proposed Mechanism	CA Source or Form	Experimental Model	Treatment	IC50/Reporting Status	Main Findings and Evidence Boundary	References
Early activity screening	Activity screening; mechanism not investigated	Biotransformed CA produced from apple-peel-derived ursolic acid using Alternaria alternata.	HepG2, MCF-7, MDA-MB-231, and Caco-2 cells.	Different CA concentrations for 48 h; the exact concentration series was not reported.	HepG2 antiproliferative activity: IC50 = 23.5 ± 2.6 μM at 48 h (mean ± SD, *n* = 8).	CA showed cytotoxic or antiproliferative activity in screening panels that included HepG2 cells. These studies established activity only and did not investigate a molecular target or cell-death programme.	[15]
		CA isolated from *Perilla frutescens* (L.) Britton var. *japonica* Hara leaves (Japanese Egoma).	HepG2, HL-60, and MCF-7 cells.	Cytotoxicity assay; the exact concentration series and exposure time were not reported in the article.	HepG2 cytotoxicity: IC50 = 37.1 μM.		[17]
		CA isolated from Red Delicious apple peels.	HepG2, MCF-7, and Caco-2 cells.	Concentration–response testing; the exact concentration series and exposure duration require confirmation from the full article.	HepG2 proliferation: EC50 = 10.56 ± 1.44 μM; HepG2 cytotoxicity: CC50 = 32.34 ± 6.30 μM.		[18]
Proliferation and cell-cycle control	P4HA2-associated growth control	Purchased CA (Beijing, Solarbio, China).	Bel-7404 and HepG2 cells.	CCK-8: 2–128 μM for 24 h. Cell-cycle and SYTOX assays: 0, 10, 20, or 30 μM for 24 h. Immunoblotting: 0, 15, or 30 μM for 24 h.	IC50 values were calculated by the authors, but numerical values were not reported in the full text.	CA reduced viability and colony formation and increased the G2/M population. P4HA2 expression decreased, but no P4HA2 genetic perturbation, rescue experiment, or direct CA–P4HA2 binding assay was performed.	[13]
	CDK19–YAP–O-GlcNAc regulation	Purchased CA (Sigma-Aldrich, St Louis, MO, USA); 20 mM methanolic stock.	Bel-7402 and Bel-7404 cells under high-glucose conditions; SMMC-7721 and SK-Hep1 were also tested. Bel-7404 subcutaneous xenografts in STZ-induced hyperglycaemic nude mice.	Cells: 0–160 μM for 24 h; mechanistic assays mainly used 40 μM for 24 h. Xenografts: 10 mg/kg/day by intratumoural injection for 20 days after tumours became palpable.	Formal IC50 not reported in the full text; 40 μM produced approximately 50% growth inhibition at 24 h under high glucose (estimated from the figure).	CA reduced CDK19 expression and YAP O-GlcNAcylation; CDK19 overexpression partly rescued the phenotype. Because CA also lowered host glucose, the xenograft study does not fully separate tumour-intrinsic from systemic metabolic effects.	[7]
	MDM2-dependent YAP destabilisation	Purchased CA (Sigma-Aldrich; CAS 4547-24-4).	SMMC-7721, Hep3B, HepG2, Huh7, and HLE cells; HepG2 subcutaneous xenografts in female nude mice.	Cells: 0, 10, 20, 40, 80, or 160 μM for 24 h; mechanistic assays mainly used 40 μM for 24 h, with selected time courses up to 96 h. Xenografts: 10 mg/kg for 28 days; route and dosing frequency were not reported.	SMMC-7721: IC50 = 41.2 μM; Hep3B: IC50 = 40.2 μM; HepG2: IC50 = 40.5 μM; Huh7: IC50 = 40.0 μM; HLE: IC50 = 41.3 μM (24 h).	CA promoted MDM2-dependent YAP degradation and inhibited tumour growth. Genetic perturbation supports pathway involvement, but direct CA binding to YAP or MDM2 was not demonstrated.	[6]
Proliferation and cell-cycle control	YAP/βTrCP regulation with actinomycin D	Purchased CA (Sigma-Aldrich, St Louis, MO, USA).	Bel-7402 and Bel-7404 (principal models), SMMC-7721, Huh7, and SK-Hep1 cells; Bel-7402 subcutaneous xenografts in athymic nude mice.	Cells: CA 0–160 μM for 24 h; mechanistic assays mainly used CA 40 μM for 24 h. ActD was used at 1 μg/mL for mechanistic assays and 5 μg/mL in the combination dose–response experiment. Xenografts: CA 10 mg/kg with or without ActD 0.02 mg/kg for 18 days; route and dosing frequency were not reported.	CA-alone IC50 = 40 μM at 24 h. ActD lowered the apparent IC50, but the combination value was not numerically reported in the full text.	ActD enhanced CA-associated YAP suppression and antitumour activity. Because ActD is not pathway-specific, the combination does not establish a selective CA–YAP interaction.	[19]
Cell death	Mitochondrial apoptosis	CA; source not reported.	HepG2 cells.	MTT: 5, 10, 20, 30, 40, 50, 60, or 70 μM for 24 h. Mechanistic assays: 30, 40, or 50 μM for 24 h.	IC50 = 40 μM at 24 h.	CA caused mitochondrial dysfunction and caspase-associated apoptosis. The evidence is limited to one hepatoblastoma-derived cell line, without rescue or in vivo validation.	[16]
	PERK–eIF2α–ATF4–CHOP-mediated ER-stress apoptosis	Purchased CA (Solarbio, China).	Bel-7402, Bel-7404, and HepG2 cells.	Apoptosis and functional assays: 0, 10, 20, or 30 μM for 24 h. Immunoblotting: 0, 15, or 30 μM for 24 h. CHOP immunofluorescence: 20 μM for 24 h. Pharmacological inhibitors were applied for 2 h before CA and maintained during the 24 h CA exposure.	IC50 not reported in the full text.	CA activated ER-stress and mitochondrial apoptotic markers; inhibitor experiments support pathway involvement. No in vivo validation or direct target-engagement assay was performed.	[3]
Cell death	Lipid-peroxidation-associated non-apoptotic cell death	Purchased CA (Sigma Chemical Co.).	Caki renal carcinoma cells were the principal model; Huh7 and SK-Hep1 were used for cross-model confirmation.	Huh7 and SK-Hep1: 10 μM for 24 h. Lipid-ROS measurements were made at 9 h; α-tocopherol was added 30 min before CA.	CA IC50 not reported in the full text.	CA induced lipid-ROS-associated non-apoptotic death across tumour models. Ferrostatin-1 and deferoxamine were ineffective, so the study does not establish ferroptosis. HCC-specific validation was limited.	[20]
	HERPUD1–MDM2–GSS–GSH ferroptosis sensitisation	Purchased CA (Solarbio, Beijing, China; product SC8500).	Bel-7402, SMMC-7721, Bel-7404, HepG2, and SK-Hep1 cells; HL-7702 non-malignant cells; subcutaneous Bel-7402 xenografts (wild-type or HERPUD1-sg1) in male athymic nude mice.	Cells: 0, 10, 20, 40, or 60 μM for 24 h. Sensitisation: 10 μM CA for 1 h before 10 μM erastin or 5 μM RSL3, followed by 24 h of treatment (lipid ROS also assessed at 10 h). Xenografts: CA 10 mg/kg and/or IKE 50 mg/kg from day 14; route and dosing frequency were not reported; tumours were monitored to day 36.	CA IC50 not reported in the full text.	Low-dose CA sensitised cells and xenografts to ferroptosis inducers through HERPUD1-dependent glutathione regulation. The evidence supports sensitisation, not ferroptosis induced by CA alone.	[12]
Migration and in vivo tumour control	VEGFR2–Src–FAK–Cdc42 migration signalling; docking plus biochemical VEGFR2 kinase inhibition, not direct cellular target engagement	Purchased CA (Sigma-Aldrich).	Huh7 cells (HepG2 and Hep3B were supplementary models); subcutaneous Huh7 xenografts in male NOD/SCID mice.	Cytotoxicity: 0–100 μM for 24 h. Migration: 0, 1, 2, 4, 8, or 16 μM for 16 h. VEGFR phosphorylation: 2 μM for 15 min. Xenografts: 5 mg/kg/day intraperitoneally for 21 days. A separate combination study used 2.5 or 5 mg/kg CA with 10 or 20 mg/kg sorafenib intraperitoneally once daily for 20 days.	Huh7 migration inhibition: IC50 ≈ 2.5 μM; Huh7 cell viability/cytotoxicity at 24 h: IC50 ≈ 50 μM; purified VEGFR2 kinase assay: IC50 = 0.95 μM.	CA inhibited migration and xenograft growth. Docking, biochemical kinase inhibition, and VEGFR2 siRNA support pathway involvement, but no biophysical binding or cellular target-engagement assay established VEGFR2 as a direct CA target. The subcutaneous model did not test invasion or metastasis.	[5]
	Orthotopic tumour control during ALPPS-associated liver regeneration	CA; source not reported.	Walker-256 rat carcinosarcoma tissue implanted orthotopically into the liver of male Sprague–Dawley rats undergoing ALPPS; this is not an HCC-specific model.	20 mg/kg intraperitoneally every 2 days from postoperative day 3 to postoperative day 15.	Not applicable (in vivo-only study).	CA reduced liver tumour growth without a clear reduction in regenerative indices. Clinical relevance is limited by the Walker-256 carcinosarcoma model, and the proposed mechanism was based mainly on immunohistochemistry without causal perturbation.	[4]

Notes: Abbreviations: ActD, actinomycin D; ALPPS, associating liver partition and portal vein ligation for staged hepatectomy; βTrCP, beta-transducin repeat-containing protein; CA, corosolic acid; CAS, Chemical Abstracts Service; CC50, half-maximal cytotoxic concentration; CCK-8, Cell Counting Kit-8; Cdc42, cell division cycle 42; CDK19, cyclin-dependent kinase 19; CHOP, CCAAT/enhancer-binding protein homologous protein; EC50, half-maximal effective concentration; eIF2α, eukaryotic translation initiation factor 2 alpha; ER, endoplasmic reticulum; FAK, focal adhesion kinase; G2/M, gap 2/mitosis phase; GSH, glutathione; GSS, glutathione synthetase; HCC, hepatocellular carcinoma; HERPUD1, homocysteine-inducible endoplasmic reticulum stress protein with ubiquitin-like domain 1; IC50, half-maximal inhibitory concentration; IKE, imidazole ketone erastin; MDM2, mouse double minute 2 homolog; MTT, 3-(4,5-dimethylthiazol-2-yl)-2,5-diphenyltetrazolium bromide; NOD/SCID, non-obese diabetic/severe combined immunodeficiency; O-GlcNAc, O-linked beta-N-acetylglucosamine; P4HA2, prolyl 4-hydroxylase subunit alpha 2; PERK, protein kinase R-like endoplasmic reticulum kinase; ROS, reactive oxygen species; RSL3, RAS-selective lethal 3; SD, standard deviation; siRNA, small interfering RNA; Src, proto-oncogene tyrosine-protein kinase Src; STZ, streptozotocin; VEGFR, vascular endothelial growth factor receptor; VEGFR2, vascular endothelial growth factor receptor 2; YAP, Yes-associated protein. In reference [18], EC50 denotes 50% of the antiproliferative effect, and CC50 denotes 50% cytotoxicity. HepG2 is hepatoblastoma-derived, and SK-Hep1 has endothelial-like characteristics; neither should be treated without qualification as a classic HCC model.

**Table 2:** Cross-model candidate mechanisms, evidence appraisal, and HCC validation priorities.

Candidate Mechanism	CA Form	Supporting Experimental Model(s)	Link to Direct HCC Evidence	Evidence Appraisal and Validation Priority	Evidence Boundary and Required HCC Validation	References
PERK-eIF2α-ATF4-CHOP ER-stress signalling	Free CA	Castration-resistant prostate cancer cells and xenografts; IRE1, PERK, and CHOP were functionally perturbed.	HCC studies support PERK/eIF2α/ATF4 activation, salubrinal rescue, and CHOP knockdown.	Genetic perturbation: Yes Pharmacological rescue: Yes Independent replication: Partial HCC-relevant in vivo validation: No Overall evidence: Moderate Validation priority: High	Cross-tumour recurrence does not establish a shared upstream trigger. Replicate PERK/CHOP dependence across HCC cell lines, organoids, and orthotopic models.	[3,21]
YAP/HERPUD1-MDM2-GSS/GSH ferroptosis axis	Free CA	Lung cancer cells; YAP loss, GSS/GSH depletion, lipid ROS, ferroptosis-associated death, and reduced EMT-related phenotypes were reported.	HCC evidence supports HERPUD1-MDM2-dependent GSS/GSH depletion and ferroptosis sensitisation, including genetic and in vivo support.	Genetic perturbation: Yes Pharmacological rescue: Partial Independent replication: Partial HCC-relevant in vivo validation: Yes Overall evidence: Moderate Validation priority: High	Upstream regulators differ between lung cancer and HCC. Do not infer a single linear pathway; test YAP, HERPUD1, MDM2, and GSS by epistasis and rescue in HCC.	[12,22]
VEGFR2/FAK-associated migration and vascular regulation	Free CA	Endothelial and lymphatic–endothelial cells and a CT-26 tumour model; endothelial proliferation, migration, tube formation, and vessel density were examined.	Huh7 migration was inhibited through VEGFR2/Src/FAK/Cdc42 signalling, with biochemical kinase and siRNA evidence.	Genetic perturbation: Yes Pharmacological rescue: No Independent replication: Partial HCC-relevant in vivo validation: Partial Overall evidence: Moderate Validation priority: High	Tumour cell migration does not establish hepatic angiogenesis or metastasis. Test hepatic endothelial co-cultures, orthotopic vessel density, invasion, metastasis, and VEGFR2/FAK perturbation.	[5,23]
STAT3-associated TAM/MDSC regulation	Free CA; CD163-targeted CA liposomes	Human macrophage, glioblastoma, ovarian cancer, and murine sarcoma models.	An ALPPS-associated orthotopic liver tumour model showed changes in macrophage, CD8-cell and vascular markers, but it used Walker-256 rat carcinosarcoma rather than an HCC-cell-derived model and did not test STAT3 causality.	Genetic perturbation: No Pharmacological rescue: No Independent replication: Partial HCC-relevant in vivo validation: Partial Overall evidence: Low Validation priority: High	Free CA and CD163-targeted delivery must be distinguished. Use immunocompetent fibrotic orthotopic HCC with STAT3 perturbation, TAM/MDSC depletion, and CD8 cell blockade.	[4,24,25,26,27,38]
AMPK-mTOR-autophagy regulation	Free CA	Gastric cancer cells, chondrocytes, hepatocytes, and cardiomyocytes; AMPK inhibition, mTOR modulation, or autophagy measurements provided functional evidence.	No direct HCC study has established AMPK-mTOR control or autophagic flux; the current link is contextual only.	Genetic perturbation: No Pharmacological rescue: Partial Independent replication: Partial HCC-relevant in vivo validation: No Overall evidence: Low Validation priority: Medium	The pathway accompanied tumour cell death or chemosensitisation in gastric cancer but cytoprotection in non-tumour cells. Measure autophagic flux, clonogenic survival, and normal-hepatocyte toxicity concurrently in HCC models.	[29,30,31,32,33,34]
TGF-β/SMAD-associated fibrosis and stromal remodelling	Free CA	NASH and hepatic stellate cell models showed lower TGF-β1/SMAD2 signalling, inflammation, collagen-associated markers, and fibrosis.	The ALPPS-associated orthotopic liver tumour study reported lower tumour TGF-β and changes in macrophage and vascular markers, but it was not based on an HCC-derived tumour model and did not include pathway perturbation.	Genetic perturbation: No Pharmacological rescue: No Independent replication: Partial HCC-relevant in vivo validation: Partial Overall evidence: Low Validation priority: High	Antifibrotic activity cannot be equated with regression of established HCC. Use immunocompetent HCC models with underlying fibrosis and assess tumour cell and stromal responses separately.	[4,28]

Notes: Abbreviations: ALPPS, associating liver partition and portal vein ligation for staged hepatectomy; AMPK, adenosine monophosphate-activated protein kinase; ATF4, activating transcription factor 4; CA, corosolic acid; Cdc42, cell division cycle 42; CD163, cluster of differentiation 163; CD8, cluster of differentiation 8; CHOP, CCAAT/enhancer-binding protein homologous protein; eIF2α, eukaryotic translation initiation factor 2 alpha; EMT, epithelial–mesenchymal transition; FAK, focal adhesion kinase; GSH, glutathione; GSS, glutathione synthetase; HCC, hepatocellular carcinoma; HERPUD1, homocysteine-inducible endoplasmic reticulum stress protein with ubiquitin-like domain 1; IRE1, inositol-requiring enzyme 1; MDM2, mouse double minute 2 homolog; MDSC, myeloid-derived suppressor cell; mTOR, mechanistic target of rapamycin; NASH, non-alcoholic steatohepatitis; PERK, protein kinase R-like endoplasmic reticulum kinase; ROS, reactive oxygen species; siRNA, small interfering RNA; SMAD, small mothers against decapentaplegic; SMAD2, SMAD family member 2; Src, proto-oncogene tyrosine-protein kinase Src; STAT3, signal transducer and activator of transcription 3; TAM, tumour-associated macrophage; TGF-β, transforming growth factor beta; VEGFR2, vascular endothelial growth factor receptor 2; YAP, Yes-associated protein. Yes indicates direct demonstration of the criterion in the cited studies. Partial indicates indirect, incomplete, cross-model, or single-study support. No indicates that the criterion was not demonstrated in the cited studies. Evidence criteria: Genetic perturbation includes knockdown, knockout, overexpression, or a comparable causal manipulation. Pharmacological rescue requires reversal or attenuation of the CA-induced phenotype by a pathway-specific intervention; simple combination treatment is not counted as rescue. Independent replication requires confirmation in an independent study; additional models within one study are partial replication. HCC-relevant in vivo validation requires mechanistic confirmation in an HCC animal model; tumour growth inhibition or marker changes alone are partial evidence. Rating standards: High evidence requires direct HCC evidence, causal perturbation or rescue, independent replication, and HCC-relevant in vivo mechanistic validation. Moderate evidence denotes direct HCC support with at least one causal test but incomplete replication or in vivo mechanistic confirmation. Low evidence denotes mainly cross-model, associative, docking-based, or marker-level support. Validation priority is rated independently: high, a decisive causal or in vivo test is needed for a mechanism central to HCC interpretation; medium, preliminary HCC confirmation is needed for a secondary mechanism; low, the mechanism is comparatively well validated or peripheral to the central HCC framework.

**Table 3:** Comparative summary of CA-related formulations, pharmacological effects, and disease models.

Formulation	Role of CA	Experimental Model	Key Formulation Feature	Pharmacological Effect	Disease Model	References
Anti-CD163 CA-containing long-circulating liposomes	Therapeutic payload	Human PBMC monocytes and monocyte-derived macrophages (MDMs) in vitro	Anti-CD163 antibody targeting of CA-loaded liposomes	Preferential STAT3 inhibition and altered cytokine expression in CD163-positive cells.	Other (non-HCC): no disease-specific tumour model; human monocyte/macrophage proof of concept.	[38]
Corosolic acid liposome (CALP) loaded with doxorubicin	Functional carrier component	Tumour cells and spheroids; 4T1 tumour-bearing BALB/c mice	CA replaced cholesterol and contributed membrane fusion, uptake, and anti-inflammatory activity	Improved doxorubicin delivery and tumour control versus cholesterol liposomes.	Other (non-HCC): breast cancer (4T1 murine mammary carcinoma).	[39]
CA-loaded kafirin nanoparticles	Therapeutic payload	MCF-7 breast cancer cells in vitro	Encapsulation efficiency 81.13%; loading 8.38%; particle size 300–400 nm; pH-dependent release	Reduced MCF-7 viability and induced apoptosis in vitro.	Other (non-HCC): breast cancer (MCF-7).	[40]
Paclitaxel-loaded CALP	Functional carrier component	Tumour cells and spheroids; 4T1 tumour-bearing BALB/c mice	CA-containing liposome intended to improve penetration and modulate the tumour microenvironment	Improved paclitaxel accumulation and antitumour activity.	Other (non-HCC): breast cancer (4T1 murine mammary carcinoma).	[57]
Size-tunable PCAL@TM micro–nano liposomes	Functional carrier component with paclitaxel/oxygen payloads	Lung-tumour-bearing mice	MMP-9-cleavable system with lung targeting, oxygen delivery, and tumour penetration	Enhanced combined treatment in lung cancer.	Other (non-HCC): lung cancer.	[58]
CA-derivative lipid nanoparticles	Derivative used as carrier material	4T1, B16/F10, and HeLa cells; intratumoural 4T1 tumour model	Cholesterol-free LNPs for mRNA/siRNA delivery	Improved RNA delivery and tumour retention with less hepatic transfection.	Other (non-HCC): breast cancer (4T1), melanoma (B16/F10), and cervical cancer (HeLa).	[59]

Notes: Abbreviations: CA, corosolic acid; CALP, corosolic acid liposome; CD163, cluster of differentiation 163; HCC, hepatocellular carcinoma; LNP, lipid nanoparticle; MDM, monocyte-derived macrophage; MMP-9, matrix metalloproteinase 9; mRNA, messenger RNA; PBMC, peripheral blood mononuclear cell; PCAL@TM, study-specific designation for the size-tunable micro–nano liposomal formulation; RNA, ribonucleic acid; siRNA, small interfering RNA; STAT3, signal transducer and activator of transcription 3. Disease models are classified from the cell lines and animal models used in the cited studies. All studies summarised here are non-HCC; reference [38] used human monocytes and macrophages in vitro without a disease-specific tumour model.

## Data Availability

No new data were created or analysed in this study. Data sharing is not applicable to this article.

## Supplementary Materials

The following supporting information can be downloaded at: https://www.mdpi.com/article/10.3390/molecules31193474/s1: Table S1A: Scopus search modules; Table S1B: Scopus search combinations and query-level yields; Table S2: Search yields across databases.

## Abbreviations

The following abbreviations are used in this manuscript:

AA	asiatic acid
ActD	actinomycin D
ADP	adenosine diphosphate
AKT	protein kinase B
ALPPS	associating liver partition and portal vein ligation for staged hepatectomy
AMPK	adenosine monophosphate-activated protein kinase
ASK1	apoptosis signal-regulating kinase 1
ATF4	activating transcription factor 4
ATP	adenosine triphosphate
Bax	Bcl-2-associated X protein
Bcl-2	B-cell lymphoma 2
βTrCP	beta-transducin repeat-containing protein
CA	corosolic acid
CALP	corosolic acid liposome
CAS	Chemical Abstracts Service
CC50	half-maximal cytotoxic concentration
CCK-8	Cell Counting Kit-8
CD163	cluster of differentiation 163
CD8	cluster of differentiation 8
Cdc42	cell division cycle 42
CDK19	cyclin-dependent kinase 19
CHOP	CCAAT/enhancer-binding protein homologous protein
CYP1A2	cytochrome P450 1A2
CYP3A4	cytochrome P450 3A4
DFO	deferoxamine
EC50	half-maximal effective concentration
eIF2α	eukaryotic translation initiation factor 2 alpha
EMT	epithelial–mesenchymal transition
ER	endoplasmic reticulum
F-actin	filamentous actin
FAK	focal adhesion kinase
G-actin	globular actin
G2/M	gap 2/mitosis phase
GSH	glutathione
GSS	glutathione synthetase
HCC	hepatocellular carcinoma
HERPUD1	homocysteine-inducible endoplasmic reticulum stress protein with ubiquitin-like domain 1
HMDB	Human Metabolome Database
HP-β-CD	hydroxypropyl-beta-cyclodextrin
IC50	half-maximal inhibitory concentration
IKE	imidazole ketone erastin
IRE1	inositol-requiring enzyme 1
JNK	c-Jun N-terminal kinase
LATS1	large tumour suppressor kinase 1
LNP	lipid nanoparticle
logD	logarithm of the pH-dependent distribution coefficient
logP	logarithm of the octanol/water partition coefficient
logS	logarithm of molar aqueous solubility
MA	maslinic acid
MDM	monocyte-derived macrophage
MDM2	mouse double minute 2 homolog
MDSC	myeloid-derived suppressor cell
MMP-9	matrix metalloproteinase 9
mRNA	messenger RNA
mTOR	mechanistic target of rapamycin
MTT	3-(4,5-dimethylthiazol-2-yl)-2,5-diphenyltetrazolium bromide
NASH	non-alcoholic steatohepatitis
NF-κB	nuclear factor kappa B
NOD/SCID	non-obese diabetic/severe combined immunodeficiency
OA	oleanolic acid
O-GlcNAc	O-linked beta-N-acetylglucosamine
OGT	O-GlcNAc transferase
p-eIF2α	phosphorylated eukaryotic translation initiation factor 2 alpha
pKa	acid dissociation constant
P4HA2	prolyl 4-hydroxylase subunit alpha 2
PBMC	peripheral blood mononuclear cell
PCAL@TM	study-specific designation for the size-tunable micro–nano liposomal formulation
PERK	protein kinase R-like endoplasmic reticulum kinase
PI3K	phosphoinositide 3-kinase
RNA	ribonucleic acid
ROS	reactive oxygen species
RSL3	RAS-selective lethal 3
SD	standard deviation
siRNA	small interfering RNA
SMAD	small mothers against decapentaplegic
SMAD2	SMAD family member 2
Src	proto-oncogene tyrosine-protein kinase Src
STAT3	signal transducer and activator of transcription 3
STZ	streptozotocin
TAM	tumour-associated macrophage
TGF-β	transforming growth factor beta
TGF-β1	transforming growth factor beta 1
UA	ursolic acid
UPLC–MS	ultra-performance liquid chromatography–mass spectrometry
VEGFR	vascular endothelial growth factor receptor
VEGFR2	vascular endothelial growth factor receptor 2
YAP	Yes-associated protein
