# Supplementary material for: Corosolic Acid in Hepatocellular Carcinoma: Molecular Properties, Evidence Boundaries, and Development Priorities
Source: Molecules. 2026 Sep 29;31(19):3474. doi: 10.3390/molecules31193474 (PMC13666057; doi:10.3390/molecules31193474)
Supplement: Supplementary file 1 [file molecules-31-03474-s001.zip › molecules-4570950-supplementary.pdf]

Supplementary Search Strategy and Search Yields

These tables document the Scopus Boolean search modules and the query-level yields used to support the literature-search methods described in Section 2. Set A captures corosolic acid and its synonyms, Set B captures hepatocellular carcinoma and related liver-cancer terminology, and Set C captures anticancer phenotypes and selected mechanistic processes. Only English-language publications were eligible at the screening stage.

Table S1A. Scopus search modules

Set	Concept	Exact Scopus query
**A**	Corosolic acid and its synonyms	TITLE-ABS-KEY(<br>"corosolic acid"<br>OR corosolic<br>OR "colosolic acid"<br>OR colosolic<br>OR "2 alpha hydroxyursolic acid"<br>OR "2alpha hydroxyursolic acid"<br>OR "2-alpha-hydroxyursolic acid"<br>OR "2α-hydroxyursolic acid"<br>OR "2α hydroxyursolic acid"<br>OR "4547-24-4"<br>)
**B**	Hepatocellular carcinoma and related liver-cancer terms	TITLE-ABS-KEY(<br>"hepatocellular carcinoma"<br>OR "hepatocellular cancer"<br>OR "hepatic carcinoma"<br>OR "hepatic cancer"<br>OR "liver cancer"<br>OR "liver carcinoma"<br>OR "liver neoplasm"<br>OR "liver tumor"<br>OR "liver tumour"<br>OR "hepatic neoplasm"<br>OR "hepatic tumor"<br>OR "hepatic tumour"<br>OR hepatoma*<br>OR hepatocarcinoma*<br>OR HCC<br>)
**C**	Anticancer phenotypes, molecular processes, and selected pathways	TITLE-ABS-KEY(<br>proliferat*<br>OR "cell viability"<br>OR "cell growth"<br>OR apoptos*

Set	Concept	Exact Scopus query
		OR caspase*<br>OR "cell cycle"<br>OR "G0/G1 arrest"<br>OR "G1 arrest"<br>OR "G2/M arrest"<br>OR migrat*<br>OR invas*<br>OR metastas*<br>OR "epithelial mesenchymal transition"<br>OR "epithelial-to-mesenchymal transition"<br>OR EMT<br>OR angiogen*<br>OR VEGF*<br>OR ferroptos*<br>OR autophag*<br>OR "reactive oxygen species"<br>OR ROS<br>OR "oxidative stress"<br>OR PI3K<br>OR AKT<br>OR mTOR<br>OR STAT3<br>OR "NF-kappaB"<br>OR "NF kappa B"<br>OR "NF-κB"<br>OR sorafenib<br>)

Search implementation. Each module was searched in the title, abstract, and keyword fields. The three modules were then combined as shown in Table S1B.

Table S1B. Scopus search combinations and query-level yields

Combination	Purpose	Records retrieved
**A AND B**	Broad retrieval of studies directly linking CA with HCC or related liver-cancer terminology.	25
**A AND B AND C**	Mechanism- and phenotype-focused subset of the direct-HCC search.	21
**A AND C**	Retrieval of CA studies reporting anticancer phenotypes or relevant molecular processes across HCC and non-HCC models.	255

Table S2. Search yields across databases

Database	Search field	A+B	A+B+C	A+C	Search date
**Web of Science Core Collection**	Topic (TS)	21	13	195	6 May 2026
**PubMed**	Title/Abstract	16	11	155	6 May 2026
**Scopus**	TITLE-ABS-KEY	25	21	255	6 May 2026
